# Ruthenium Complexes in Protein Science: Heading a New Generation of Therapeutic and Analytical Tools

**DOI:** 10.1002/anie.202514884

**Published:** 2025-12-02

**Authors:** Liyan Zhang, Yun Chen, Sylvestre Bonnet

**Affiliations:** ^1^ MOE International Joint Research Laboratory on Synthetic Biology and Medicines School of Biology and Biological Engineering South China University of Technology Guangzhou 510006 P.R. China; ^2^ Leiden Institute of Chemistry Universiteit Leiden Einsteinweg 55 Leiden 2333 CC Netherlands; ^3^ School of Chemistry Chemical Engineering and Biotechnology Nanyang Technological University 62 Nanyang Drive Singapore 637459 Singapore

**Keywords:** Catalytic labelling, Chemosensor, Protein binding, Protein labelling, Ruthenium complex

## Abstract

Ruthenium complexes are distinguished by their versatile coordination chemistry, redox behaviors and photochemical properties. In recent years, this tunability has enabled precise control over their interactions with peptides and proteins, opening new avenues in therapeutic and analytical applications. This minireview provides a concise overview of the growing role of ruthenium complexes in protein science, with a focus on latest advances in protein targeting, inhibition, labelling, and sensing.

## Graphical abstract

This review presents the latest advances in the development of ruthenium complexes within the field of protein science, highlighting their potential applications in biomedical and bioanalytical fields.

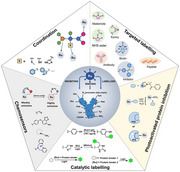

## 1 Introduction

Since the clinical approval of cisplatin for cancer treatment in 1978, metallodrugs have become a prominent focus in oncological research.^[^ ^1^ ^]^ Over the past decades, significant progress has been made in the exploitation of alternative metal centres for anticancer treatment and diagnosis, such as gold,^[^ ^2^ ^]^ palladium,^[^ ^3^ ^]^ iron,^[^ ^4^ ^]^ ruthenium,^[^ ^5^ ^]^ or iridium.^[^ ^6^ ^]^ Owing to their versatile modes of action and highly tuneable coordination environments, metal–organic complexes have emerged as promising platforms for cancer therapy and molecular diagnostics. Moreover, many metal‐based prodrugs can be engineered for stimuli‐responsive activation under specific physiological conditions such as pH,^[^ ^7^ ^]^ redox,^[^ ^8^, ^9^ ^]^ light,^[^ ^10^ ^]^ ultrasound,^[^ ^11^ ^]^ and X‐ray.^[^ ^12^ ^]^ Among these metals, ruthenium (Ru) has attracted considerable attention in inorganic chemical biology and bioinorganic medicinal chemistry.^[^ ^13^, ^14^, ^15^ ^]^ Unlike square‐planar d^8^ platinum(II) compounds, Ru(II) complexes are d^6^ and typically hexacoordinated, which provides them with a three‐dimensional (3D) octahedral architecture. The diverse ligands available for octahedral centres enable precise tuning of their properties. Recently, this versatility has drawn increasing attention in bioinorganic chemistry, especially for designing new octahedral Ru complexes that interact with biomolecules.

Proteins and peptides are essential molecular building blocks and functional entities in biology, making them prime targets for therapeutic and analytical applications. Octahedral Ru complexes, with their unique electronic properties, can engage in diverse interactions with these biomolecules. First, they are redox active and can exist in multiple oxidation states (e.g., Ru(II) and Ru(III)), which enables the metal centre to undergo redox cycling. This characteristic can be leveraged in catalysis on biological substrates, targeting cellular processes like oxidative stress and mitochondrial dysfunction.^[^ ^16^, ^17^ ^]^ Second, Ru complexes can be functionalised with a wide range of ligands, allowing fine‐tuning of their binding affinity and selectivity for specific protein targets.^[^ ^18^ ^]^ Third, many Ru complexes exhibit luminescence or undergo photochemical activation, enabling real‐time imaging of protein localisation, dynamics, and interactions, as well as facilitating selective modification or crosslinking of protein residues and targeted inhibition or damage of protein for therapeutic applications.^[^ ^19^ ^]^

**Scheme 1:**
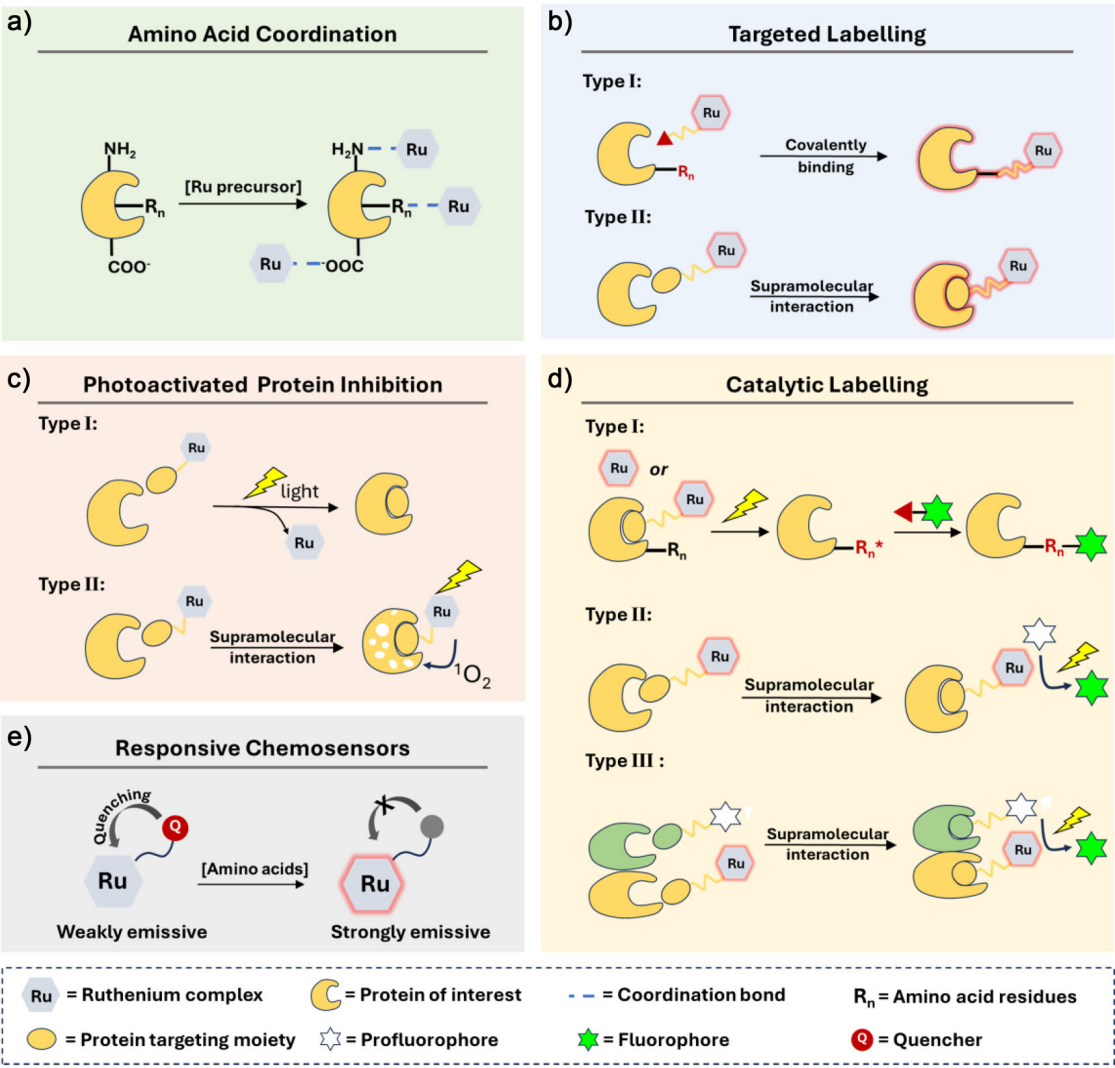
Schematic overview of the protein binding, labelling, and inhibition mechanisms described in this review for Ru complexes.

Although the structural information available from X‐ray structures about the interactions between Ru complexes and protein residues has been reviewed not so long ago,^[^ ^20^, ^21^ ^]^ a broader perspective on the role of Ru chemistry in protein science is still lacking, which this minireview aims to address. We highlight latest advances in the application of Ru complexes for protein modification, inhibition, and labeling, focusing on five main aspects: 1) coordination with amino acid residues; 2) targeted protein labeling; 3) photoactivated protein inhibition; 4) catalytic protein modification and labelling; and 5) development of Ru‐based chemosensors responsive to amino acids (Scheme 1). We are confident that this focused, yet concise review will offer valuable insights into the emerging role of Ru complexes in protein science and serve as a source of inspiration for researchers at the intersection of inorganic chemistry, protein science, and their healthcare applications.

## 2 Amino Acid Coordination

Metal elements are commonly found as essential cofactors in natural proteins and play an indispensable role in many process such as catalysis, protein storage and transport, infectious disease, etc.^[^ ^22^ ^]^ These ions typically interact with the side chains of amino acid residues in proteins; particularly common metal‐binding residues are the imidazole group of histidine (His), the thiolate group of cysteine (Cys), and the carboxylate group of aspartate (Asp).^[^ ^23^ ^]^ Nevertheless, due to the vast diversity of the metalloproteome, virtually all amino acid residues are potential ligands toward metal centres. The peptide backbone can also contribute to metal coordination through deprotonated amide nitrogen and amide carbonyl oxygen atoms (Scheme 1a). However, conjugates of Ru complexes involving coordination bonds between Ru and proteins or peptides remain relatively underexplored.^[^ ^24^, ^25^ ^]^

**Figure 1:**
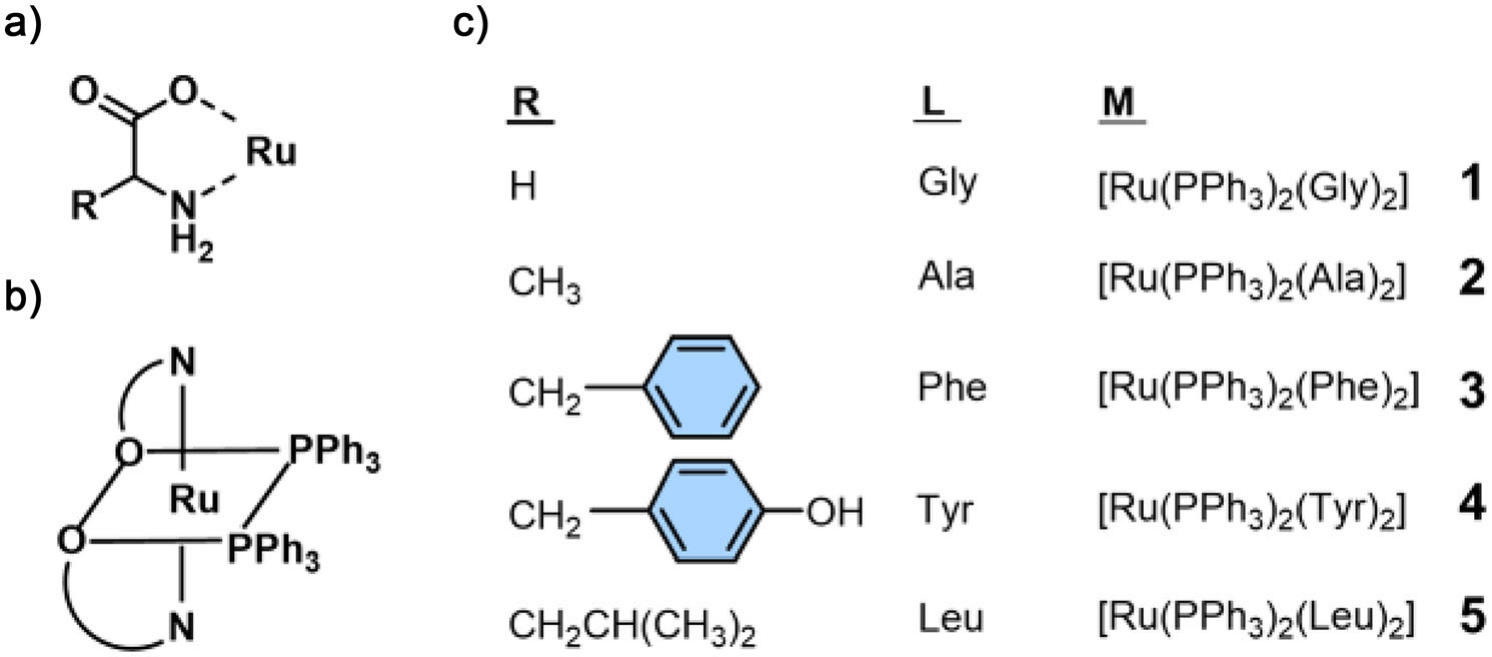
Structure of **1–5** formed by the reaction of deprotonated α‐amino acids with the precursor [Ru(PPh_3_)_3_Cl_2_]. Adapted with permission.^[^ ^26^ ^]^ Copyright 1999, Elsevier.

In an early study, Bhattacharya et al. reported the reaction of several α‐amino acids (HL) with the precursor [Ru(PPh_3_)_3_Cl_2_] in the presence of a base (triethylamine), affording a family of complexes of the type [Ru(PPh_3_)_3_(L)_2_] (Figure 1a).^[^ ^26^ ^]^ Upon deprotonation of the ammonium group, L^–^ can coordinate to Ru through the neutral amine and the carboxylate group, acting as bidentate N, O‐donors to form five‐membered chelate rings (Figure 1b). Five amino acids including glycine (Gly), alanine (Ala), phenylalanine (Phe), tyrosine (Tyr), and leucine (Leu) were successfully coordinated to the metal centre (Figure 1c, compounds **1–5**), showing that this bidentate coordination mode was applicable to a wide variety of free amino acids.

**Figure 2:**
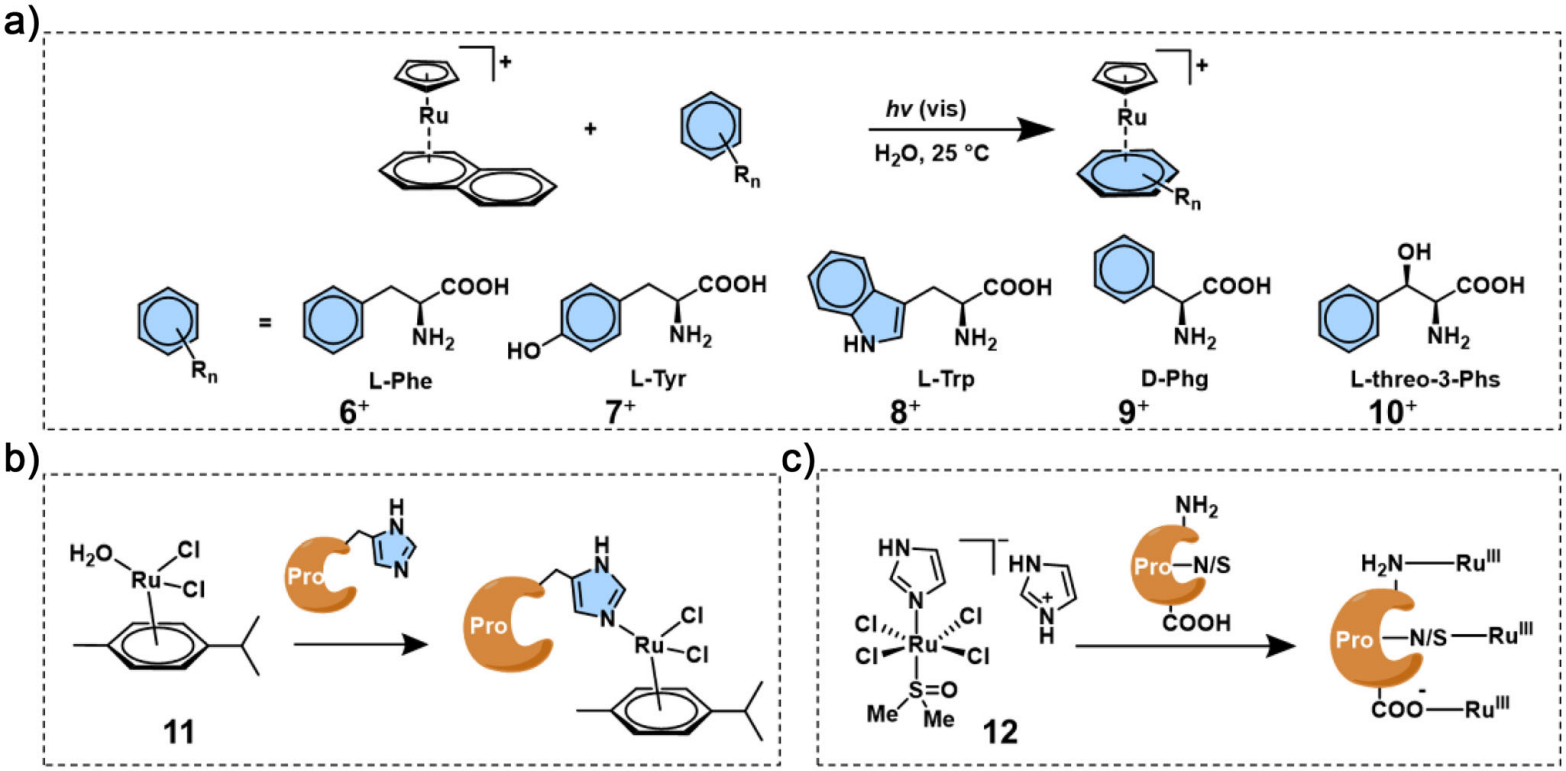
a) Structure of **6** ^+^‐**10** ^+^ obtained by reaction of [Ru(*η^6^*‐C_10_H_8_)(Cp^−^)]^+^ (Cp^–^ = C_5_H_5_^–^) with aromatic amino acids under visible‐light irradiation.^[^ ^28^ ^]^ b) Structure of **11** and its reaction with Nε of the imidazole ring of the lysozyme protein.^[^ ^31^ ^]^ c) Structure of **12** and the Ru^III^‐BSA (bovine serum albumin) adducts.^[^ ^32^ ^]^

Free amino acids are generally considered the principal currency of protein metabolism in multicellular organisms; however, their concentrations are relatively low compared with those in the protein‐bound form.^[^ ^27^ ^]^ Thus, the side chains of amino acids in proteins and peptides are of broader interest for the binding to Ru complexes. In the work reported by Kudinov et al.,^[^ ^28^ ^]^ the sandwich complexes [Ru(*η^6^*‐C_10_H_8_)(Cp^−^)]^+^ (Cp^−^ = C_5_H_5_^–^) reacted with the side chains of aromatic amino acids *L*‐phenylalanine, *L*‐tyrosine, *L*‐tryptophan, *D*‐phenylglycine, and *L*‐threo‐3‐phenylserine under visible‐light irradiation, affording [Ru(η^6^‐amino acid)(Cp^−^)]^+^ complexes in near‐quantitative yields (Figure 2a, **6** ^+^ **−10** ^+^). Such π coordination with aromatic amino acids is highly selective, mild, and stable, making it useful for peptide labelling and related applications.^[^ ^29^ ^]^ For example, in a subsequent study, Buglyó and coworkers successfully attached a (Cp^−^)Ru(peptide) fragment to one side of the histidyl–alanyl–valinyl (HAV) peptide sequence via η^6^‐coordination with the Tyr residue, while the other side of the peptide was covalently attached to a gallium centre for use in radio‐imaging.^[^ ^30^ ^]^

His, on the other hand, is one of the most important amino acids for metal binding in proteins. The imidazole motif of the His residue has commonly served as ligand for Ru. For example, Sadler and coworkers obtained the first crystal structure of a lysozyme protein coordinated to a pianostool Ru complex [Ru(η^6^‐*p*‐cymene)(H_2_O)Cl_2_] (Figure 2b, **11**), showing selective coordination of the Ru with the N_ε_ of the imidazole ring of lysozyme‐His15.^[^ ^31^ ^]^ But Ru can also attach to His side chains of a protein as modification tool. For instance, [Ru(NH_3_)_5_(H_2_O)]^2+^ was first employed as a His‐selective protein‐modification reagent for ribonuclease A,^[^ ^33^ ^]^ followed by other Ru complexes.^[^ ^34^ ^]^ In a later work, Lay and coworkers found that NAMI‐A (**12**), one of the few Ru compounds that reached clinical trials, rapidly converted into Ru^III^‐bovine serum albumin (BSA) adduct in cell‐culture medium. The protein ligand donors, including sulfur groups, amine/imine and carboxylato groups, could replace all original monodentate ligands of **12**, i.e., Cl^–^, imidazole and dimethyl sulfoxide (DMSO), affording a naked Ru^3+^ centre bound to protein. (Figure 2c). This ligand exchange, confirmed by X‐ray crystallography, explains both the high anti‐metastatic efficacy and low toxicity of NAMI‐A,^[^ ^32^ ^]^ emphasising the importance of understanding the exchange reactions of Ru compounds in biological environments.^[^ ^35^ ^]^

**Figure 3:**
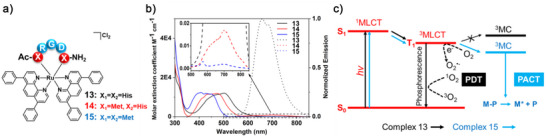
Structure of **13**–**15** a), their absorption (plain lines) and emission (dashed lines) spectra in MilliQ H_2_O b), and their Jablonski diagram c). Reproduced from Ref. [38] under the Creative Commons Attribution 4.0 International License (CC BY 4.0).

Within sulfur ligands, cysteine residues can also act in theory as monodentate thiol ligands for Ru(II) polypyridyl complexes, but Bonnet and coworkers have demonstrated the difficulty of such syntheses and the thermal instability of the resulting coordination complexes, notably towards O_2_.^[^ ^36^ ^]^ Methionine residues are more versatile ligands for Ru complexes and form thermally more stable, and photochemically more labile, coordination bonds.^[^ ^37^ ^]^ Recently, the Bonnet group^[^ ^38^ ^]^ compared the coordination of terminal His and Met residues in RGD‐containing pentapeptides to [Ru(Ph_2_phen)_2_Cl_2_], to synthesise integrin‐targeted Ru(II)‐peptide conjugates. The pentapeptides coordinated to Ru via two coordination bonds: Ru‐(κ^N^‐His) or Ru‐(κ^S^‐Met) (Figure 3a). Interestingly, bis‐His compound **13** was much more emissive and possessed higher singlet oxygen (^1^O_2_) generation quantum yields under visible light excitation than Met‐containing compounds **14** and **15** (Figure 3b). It turned out that different types of ligands from the side chain of the protein would endow different photochemical properties in the final conjugates, making **13** a photodynamic therapy (PDT) compound and **15** a photoactivated chemotherapy (PACT) compound (Figure 3c). As the use of Ru complexes in PDT and PACT has been broadly reviewed,^[^ ^39^, ^40^, ^41^ ^]^ we will not cover it here. Rather, we emphasise how protein coordination sites can modulate the photochemical properties of Ru complexes.

## 3 Targeted Labelling

In addition to direct coordination of amino acid residues to Ru, several alternative strategies have been developed to achieve protein binding and targeting using Ru complexes. First, a wide family of coordinatively saturated Ru complexes was shown by Meggers's group to act as inhibitors of enzymatic activity.^[^ ^42^, ^43^, ^44^ ^]^ The selective inhibition of these enzymes, e.g., proteases or kinases, is of great interest in cancer treatment. Like organic protein inhibitors, Ru‐based inhibitors have strict requirements on the fit between the structure of the metal compound and the shape and functionalisation of the protein pocket. A more straightforward approach is to conjugate the Ru complex with various protein‐targeting organic ligands (e.g., antibodies, peptides or small molecules) that are known to bind to specific protein receptors on the surface or in the intracellular region of cells, for example, integrins.^[^ ^45^ ^]^ This strategy allows for the selective binding of Ru‐containing inhibitors to the active site, thereby minimising off‐target effects. Comprehensive reviews about these topics have been reported;^[^ ^46^ ^]^ hence, we will not discuss it here in detail.

**Figure 4:**
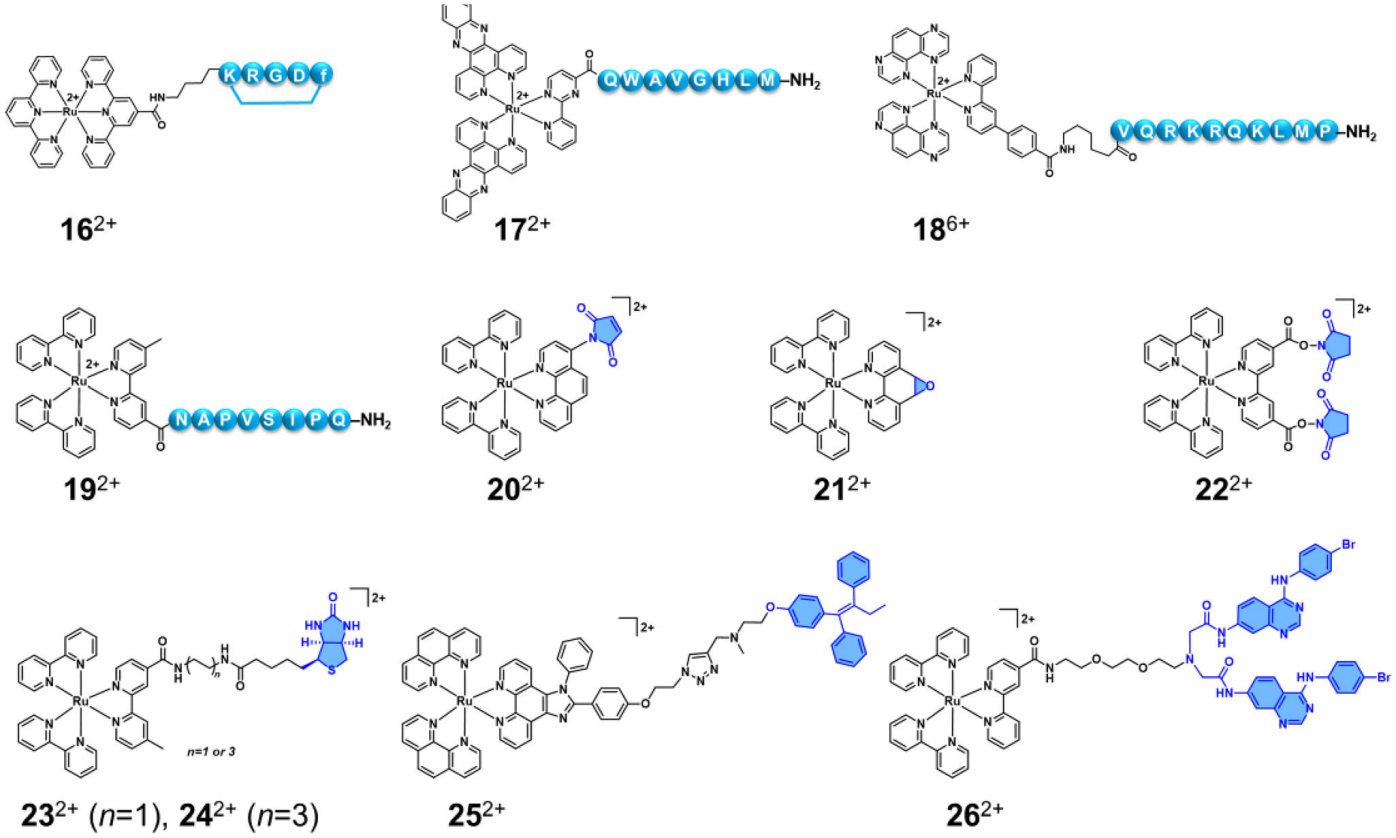
Structures of Ru complexes **16** ^2+^‐**26** ^2+^ for protein‐targeted labelling. The charges of the peptide conjugates may depend on the protonation state of the peptide and are not indicated for clarity.

Next to Ru complexes capable of inhibiting proteins, those with phosphorescent properties have attracted attention in bioimaging and biosensing owing to their long‐lived excited states, large Stokes’ shifts, high photostability, and tuneable emission.^[^ ^14^ ^]^ When coupled with targeting motifs, these conjugates can localisee to specific proteins (Scheme 1b). They generally consist of three components: an emissive Ru polypyridyl complex, a linker (optional), and a functional motif for selective protein recognition. A wide variety of functional groups including nucleotides, carbohydrates, lipids, inhibitors, and peptides, have been attached to Ru complexes for targeted delivery.^[^ ^18^, ^47^ ^]^ For protein targeting, most efforts have focused on peptides and small molecules, the latter functioning either through covalent binding or non‐covalent inhibition via multiple weak interactions (Figure 4).

Peptides, defined as short chains of 2–50 amino acids linked by amide bonds, often represent minimal functional domains for larger proteins.^[^ ^48^ ^]^ The covalent conjugation of peptides with luminescent Ru complexes has been widely reported due to their easy synthesis, low toxicity, and high biological specificity. A well‐known example is the cyclic peptide *cyclo*‐RGDfK (**16** ^2+^), which has been applied widely to target integrin proteins involved in cell adhesion.^[^ ^49^, ^50^, ^51^ ^]^ By a similar strategy, other peptide sequences were also reported to conjugate with Ru(II) polypyridine complexes, including the bombesin receptor (BBR) targeted sequence QWAVGHLM (**17** ^2+^) reported by Gasser and coworkers,^[^ ^52^ ^]^ or the importin‐targeted sequence VQRKRQKLMP **(18** ^6+^) reported by Keyes and coworkers.^[^ ^53^ ^]^ One latest report was from Das and coworkers,^[^ ^54^ ^]^ who described the conjugation of a microtubule‐ (MT‐) targeted octapeptide NAPVSIPQ (NAP) to [Ru(bpy)_3_]^2+^ (**19** ^2+^). This compound showed higher binding affinity over the free NAP peptide toward β‐tubulin, which was attributed to electrostatic interactions between the microtubule and the cationic Ru(II) centre. Other peptide sequences were also reported for the application of targeted therapy and diagnosis by Ru complex,^[^ ^45^, ^55^, ^56^ ^]^ which will not be discussed here in detail.

In addition to peptide‐based approaches, more classical covalent strategies have been employed to attach Ru complexes to amino acid side chains.^[^ ^57^ ^]^ Among these, maleimide chemistry has been widely used due to its rapid and selective thiol–Michael addition reactivity toward Cys residues. More than 20 years ago, Lakowicz and colleagues reported a maleimide‐functionalised [Ru(bpy)_2_(phen)]^2^⁺ complex (phen = 1,10‐phenanthroline) that covalently bound to human serum albumin, immunoglobulin G (IgG), and β‐galactosidase (**20** ^2+^), demonstrating the compatibility of this widely used method to Ru complexes.^[^ ^58^ ^]^ More recently, other electrophilic ligands,^[^ ^59^ ^]^ such as 5,6‐epoxy‐5,6‐dihydro‐1,10‐phenanthroline ligand, have also enabled selective sulfhydryl conjugation of Ru complexes to Cys (**21** ^2+^).^[^ ^60^ ^]^ For lysine (Lys) residues, which are typically more abundant and more accessible on protein surfaces than Cys residues,^[^ ^61^ ^]^ the classical functional group N‐hydroxysuccinimide (NHS) ester was also shown as early as 1995 to allow covalent attachment of Ru complexes to proteins. Lakowicz et al then described the first preparation of NHS ester‐functionalised [Ru(bpy)_3_]^2^⁺ derivatives for probing protein rotational dynamics (**22** ^2+^),^[^ ^62^ ^]^ but it has been used throughout the years up to Xing's recent demonstration of this strategy to develop a Ru‐based electrochemiluminescence probe that can be used for an immunoassay or a nucleic acid assay.^[^ ^63^ ^]^ It is worth noting that although many electrophiles are well known for covalently targeting nucleophilic amino acids in proteins, their use in ruthenium complexes remains largely unexplored.^[^ ^64^ ^]^

Biotin, also known as vitamin B7 or vitamin H, is a small, water‐soluble molecule that plays a vital role in cellular metabolism as a coenzyme for carboxylase enzymes. In biochemical applications, biotin is best known for its exceptionally strong and specific non‐covalent interaction with avidin, streptavidin, or neutravidin proteins (K_d_ ≈ 10^−15^ M).^[^ ^65^ ^]^ Notably, the intrinsic photophysical properties of the biotinylated molecules have mostly remained.^[^ ^66^, ^67^ ^]^ In 2004, Lee and coworkers developed two Ru‐based luminescent probes for avidin by attaching a biotin moiety to a Ru(II) polypyridine luminophore via two linkers with different lengths (**23** ^2+^ and **24** ^2+^).^[^ ^68^ ^]^ It is worth noting that several other biotinylated Ru complexes have also been developed, such as Chao and coworkers,^[^ ^69^ ^]^ Sousa and coworkers,^[^ ^70^ ^]^ Bonnet and coworkers,^[^ ^71^ ^]^ and Chen and coworkers.^[^ ^72^ ^]^

Finally, protein‐targeted small inhibitors play a critical role in modern drug discovery and therapeutic development. These compounds are designed to selectively bind to specific proteins—often enzymes, receptors, or signalling molecules—to modulate their activity, typically by blocking active sites or interfering with protein–protein interactions.^[^ ^73^, ^74^, ^75^ ^]^ Due to their low molecular weight, they can be covalently attached to multiple substrates as a targeting cargo to enhance specificity, efficacy, and duration of action. In 2018, Peng and coworkers reported the first case of a Ru(II) polypyridyl complex targeted to the estrogen receptor (ER, **25** ^2+^) through the connection of tamoxifen to the luminescent [Ru(phen)_2_(phenimi)]^2+^ complex via a triazole‐containing linker.^[^ ^76^ ^]^ Tamoxifen is a selective estrogen receptor modulator (SERM), it was shown that the attachment of this modulator enhanced the targeting of the conjugate in ER‐positive cells. Similarly, Georgiades and colleagues developed a series of conjugates in which one or two epidermal growth factor receptor tyrosine kinase inhibitors (EGFR‐TKIs) were attached to the [Ru(bpy)_3_]^2^⁺ core.^[^ ^77^ ^]^ The bis‐conjugate, referred to as **26** ^2+^ in Figure 4, achieved both subcellular imaging and EGFR inhibition, and due to its strong potential for crossing the BBB, the conjugate represents a valuable candidate for further development in glioblastoma imaging, therapy, and disease monitoring.^[^ ^77^ ^]^ Overall, the multiple examples described in this section demonstrate that coordination bonds involving Ru^2+^ ions are often stable enough to allow protein tagging and labelling with Ru compounds.

## 4 Photoactivated Protein Inhibition

As indicated above, luminescent Ru(II) complexes have been widely used for the labelling and imaging of cells, and some have also been successfully applied in targeted photodynamic therapy (PDT).^[^ ^41^ ^]^ Beyond these applications, Ru complexes are also promising candidates in PACT, also referred to as photochemotherapy (PCT).^[^ ^78^, ^79^ ^]^ This therapeutic strategy uses light to release biologically active ligands and metal centres from Ru‐based non‐toxic prodrugs, which allows precise control over when and where a drug becomes biologically active.^[^ ^40^ ^]^ Three designs of such prodrugs have been proposed based on the function of the ligand‐ and metal‐based photoproduct. First, prodrugs have been prepared where the ligand is non‐toxic and serves as a protective “cage”. Light activation generates an unsaturated Ru species capable of binding to biological targets such as proteins or DNA. Second, the ligand serves as the active drug (Scheme 1c **, Type I**), its cytotoxic properties are caged in the dark by the inert Ru(II) complex. Photoinduced ligand release results in the delivery of the active organic drug to its biological target. Third, dual action systems were suggested where both the photoreleased organic ligand and the Ru‐based photoproduct are biologically active, leading to potentially synergistic or multi‐targeted therapeutic effects.

So far, protein inhibitors are among the most widely employed ligands in PACT, enabling light‐controlled, selective inhibition of target proteins. One of the most extensively studied Ru carriers for photoactivated ligand release is the [Ru(bpy)_2_(L_1_)(L_2_)]^2+^, pioneered by Etchenique,^[^ ^80^ ^]^ Turro^[^ ^81^ ^]^ and Glazer^[^ ^82^ ^]^ et al. At early stages, it was demonstrated to be able to photorelease small and labile ligands such as 4‐aminopyridine, neurotransmitting amines, and sterically hindered pyridines. The resulting photoproduct, [Ru(bpy)_2_(H_2_O)_2_]^2^⁺, was recognised for its DNA‐binding properties and considered as a potential warhead for nucleic acid targeting. Subsequent work expanded the scope of releasable ligands to include a variety of imidazole‐, pyridine‐ and nitrile‐based inhibitors, enabling this Ru scaffold to become a dual‐action agent that combines protein inhibition from the photoreleased ligands, with DNA binding by the metal‐based photoproduct.^[^ ^83^, ^84^ ^]^

**Figure 5:**
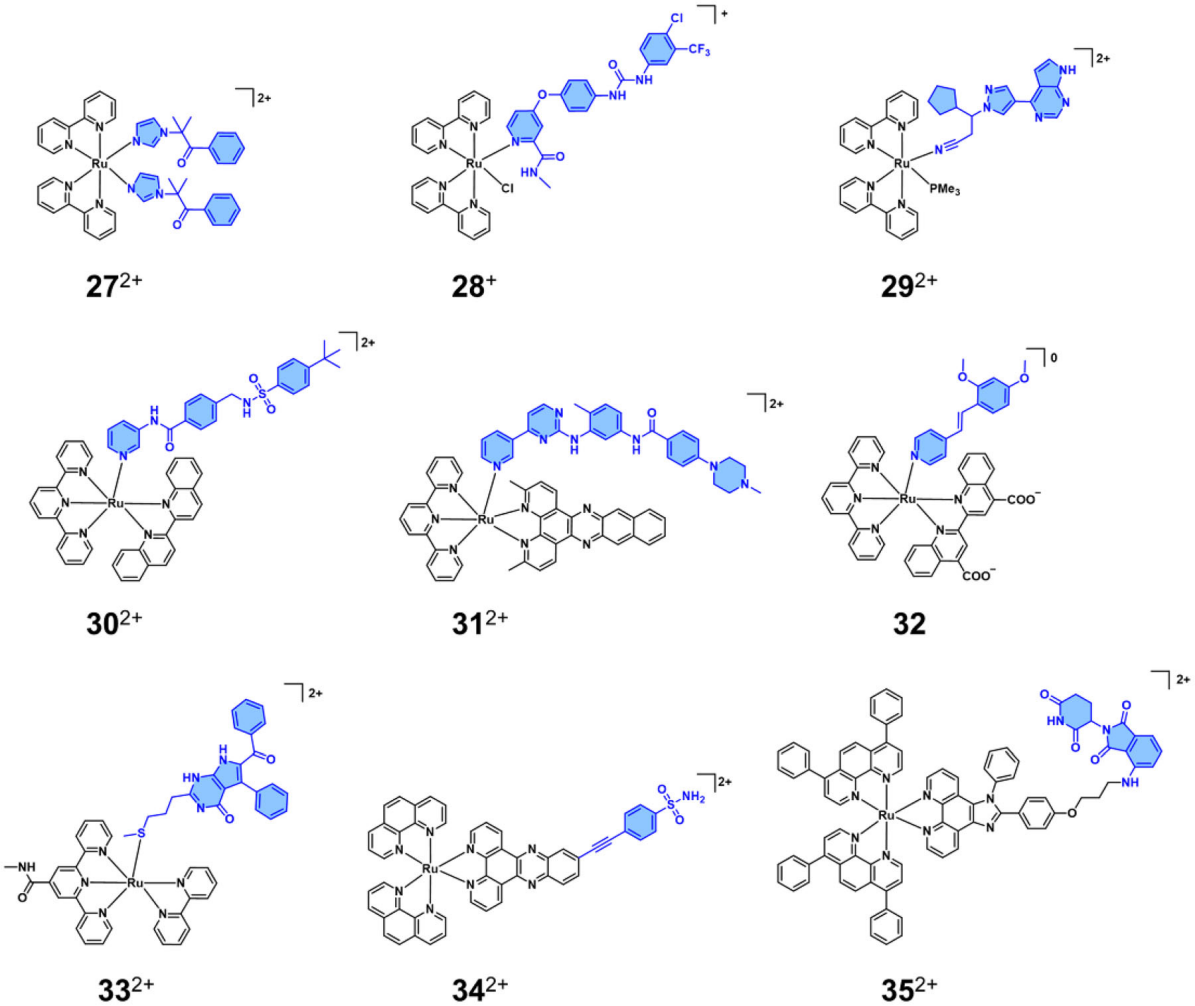
Structures of Ru complexes **27** ^2+^‐**35** ^2+^ used for photoactivated protein inhibition.

For example, Glazer and coworkers successfully employed the [Ru(bpy)_2_(L)_2_]^2+^ scaffold to photorelease cytochrome P450 inhibitors upon blue light irradiation (470 nm) (**27** ^2+^ in Figure 5). The photoactivated complex exhibited dual functionality, inducing DNA damage through the Ru centre while inhibiting P450 enzymes via the liberated ligand.^[^ ^85^ ^]^ Furthermore, the photorelease of two FDA‐approved drugs, i.e., sofafenib (**28** ^2+^) and ruxolitinib (**29** ^+^) from Zhang and coworkers ^[^ ^86^ ^]^ and Etchenique and coworkers ^[^ ^87^ ^]^ were also confirmed using this strategy. Notably, sorafenib is a multi‐kinase inhibitor, whereas ruxolitinib selectively inhibits Janus kinases (JAKs).

Another class of Ru scaffolds commonly employed in PACT is sterically hindered Ru photocaging scaffolds, primarily based on the [Ru(tpy)(N‐N)]^2+^, where tpy = 2,2′;6′‐2′′‐terpyridine and N‐N is a sterically bipyridine chelate, and L is a monodentate ligand.^[^ ^88^ ^]^ Compared to the [Ru(bpy)_2_(L)_2_]^2^⁺ platform, these complexes absorb light in the red region of the spectrum, which is more desirable for biological applications. In 2017, Bonnet and coworkers reported the red‐light‐activated (625 nm) photorelease of a nicotinamide phosphoribosyltransferase (NAMPT) inhibitor (named STF31) from complex [Ru(tpy)(biq)(STF31)]^2+^ (biq = 2,2′‐biquinoline, **30** ^2+^),^[^ ^89^ ^]^ Notably, the red‐light‐activated cytotoxicity of **30** ^2+^ under hypoxia was comparable to that observed under normoxia, highlighting the superiority of this strategy over traditional photodynamic therapy approaches, which often suffer from low oxygen concentrations.

The structure of the N‐N ligand could be finely tuned to endow the complexes with unique properties. In 2018, Turro and coworkers demonstrated the release of FDA‐approved tyrosine kinase inhibitor, i.e., imatinib, from [Ru(tpy)(N‐N)(imatinib)]^2+^ complexes, (**31** ^2+^, where *N*‐*N* = 3,6‐dimethylbenzo[*i*]dipyrido[3,2‐*a*:2′,3′‐*c*]phenazine (Me_2_dppn)).^[^ ^90^ ^]^ Most importantly, **31** ^2+^ demonstrated a dual‐action therapeutic mechanism, effectively combining PACT and PDT capabilities, due to the presence of benzo[i]dipyrido[3,2‐a:2′,3′‐c]phenazine (dppn), and an effective photorelease of Imatinib. In another work from Glazer and coworkers,^[^ ^91^ ^]^ a pyridyl ring‐substituted CYP1B1 cytochrome P450 1B1 enzyme (CYP1B1) inhibitor was subsequently “caged” to a Ru(II) scaffold, which carried the [2,2′‐biquinoline]‐4,4′‐dicarboxylic acid (bca) ligand (**32**). The inclusion of the bca ligand enhanced the water solubility of the complex while maintaining an extended absorption profile,^[^ ^92^ ^]^ with the absorption tail reaching up to 700 nm.

Without using a sterically hindered ligand, photolabile Ru‐thioether coordination can also allow successful photorelease of a cytotoxic ligand L in the [Ru(tpy)(N‐N)(L)]^2+^ scaffold. For example, Bonnet and coworkers reported the photorelease of a thioether‐containing microtubule inhibitor with green light irradiation (530 nm).^[^ ^93^ ^]^ Recently, the same group found out that introducing a simple methylamide group in the 4′ position of the terpyridine ligand could dramatically accelerate the red‐light‐activated ligand release (**33** ^2+^).^[^ ^94^ ^]^

Next to PACT, photodynamically active Ru complexes can also be used for the selective photo‐inactivation of proteins via a technique called chromophore‐assisted light inactivation (CALI).^[^ ^95^ ^]^ In this approach, protein‐targeting antibodies or small molecules are linked to a Ru photosensitizer. Light activation of the conjugate produces singlet oxygen with a short diffusion range (∼40–80 Å),^[^ ^96^ ^]^ allowing localised and specific inactivation of the target protein (Scheme 1c **, Type II**). Representative examples of this strategy have been reported by Kodadek and coworkers,^[^ ^97^, ^98^ ^]^ who first conjugated a vascular endothelial growth factor receptor 2 (VEGFR2) antagonist to [Ru(bpy)_3_]^2+^, which irreversibly inhibited the activation of VEGFR2 upon visible light activation. More recently, Hao and coworkers reported the light‐induced degradation of carbonic anhydrase IX (CAIX) by conjugating a CAIX inhibitor to a Ru(II) complex (**34** ^2+^),^[^ ^99^ ^]^ while Chao and coworkers demonstrated two‐photon–activated degradation of the E3 ubiquitin ligase cereblon (CRBN) through attachment of a CRBN‐targeting ligand to a Ru(II) scaffold (**35** ^2+^).^[^ ^100^ ^]^ Overall, the excellent thermal stability of Ru compounds, combined with their unique visible‐light absorption and finely tuneable photochemical properties, has enabled efficient light‐induced protein inhibition or inactivation in cancer cells, offering promising phototherapeutic potential in oncology.

## 5 Catalytic Labelling

The broad utility of protein bioconjugates has driven the demand for innovative and diverse strategies for site‐selective protein modification. Among these, transition metal‐catalysed reactions, which are widely employed in organic synthesis, offer a powerful and versatile platform for expanding the bioconjugation toolkit.^[^ ^101^ ^]^ The use of organometallic intermediates has significantly advanced synthetic chemistry by enabling previously challenging bond‐forming reactions.^[^ ^102^ ^]^ Among these, several Ru‐(photo)catalysed pathways have been applied for protein modification and labelling under biological conditions. These include three main types noted type I‐III in Scheme 1d.

**Figure 6:**
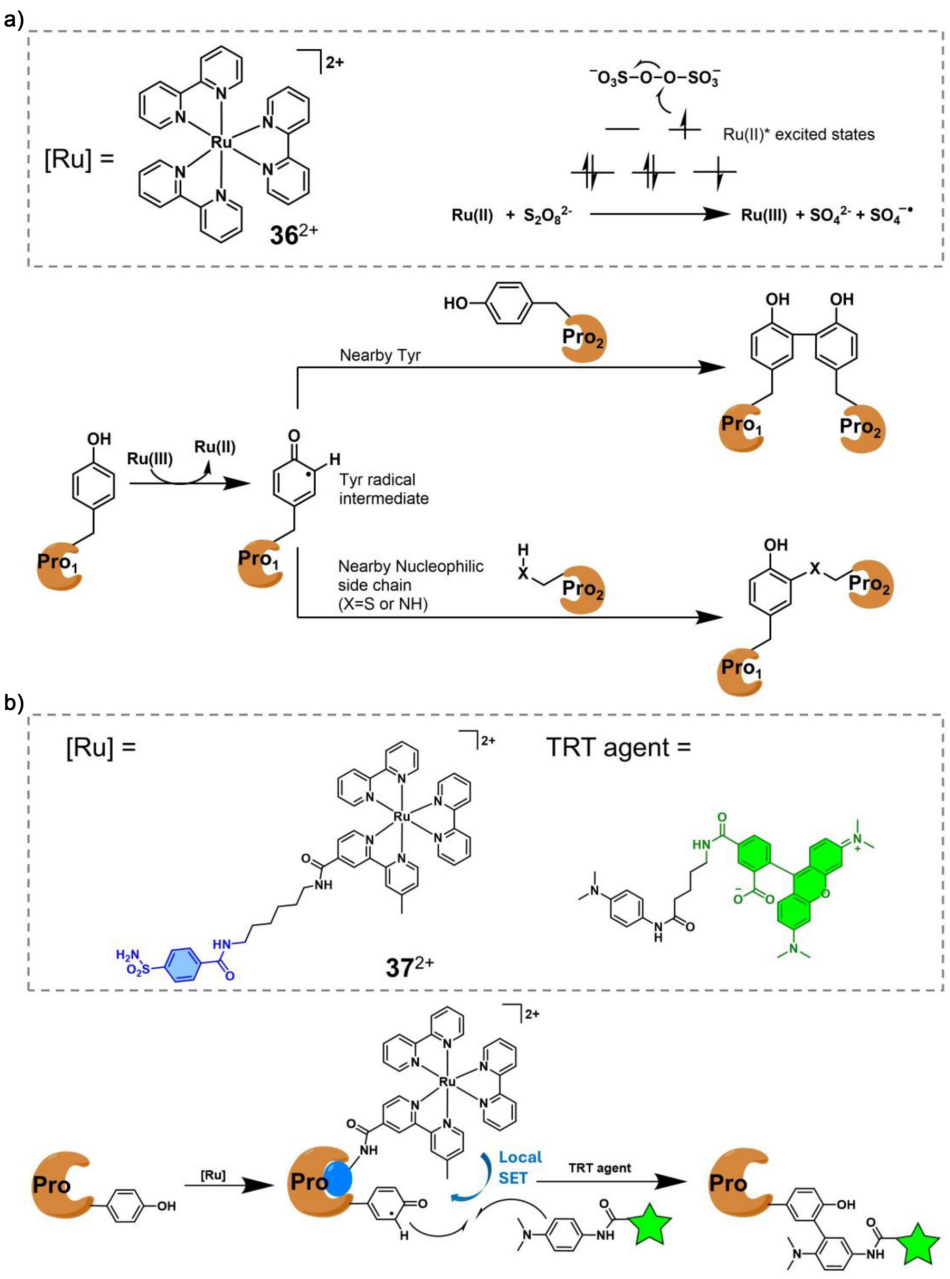
a) Protein cross‐linking reaction induced by **36** ^2+^ and visible light.^[^ ^106^ ^]^ Copyright 1999, National Academy of Sciences. b) **37** ^2+^ functioned as a local single‐electron transfer (SET) catalyst for Tyr labelling. Adapted with permission.^[^ ^108^ ^]^ Copyright 2023, Wiley‐VCH.

In type I, Ru polypyridyl complexes selectively oxidise, upon photoexcitation, amino acid residues (*R*_n_) such as tyrosines, to generate transient radicals; these radicals can subsequently be trapped by probe‐bearing reagents to yield labelled protein conjugates. Complex **36** ^2+^, also referred to as [Ru(bpy)_3_]^2+^, plays a prominent role in this field. It possesses excellent photophysical properties including strong blue light absorption, long‐lived excited states, as well as an energy‐rich excited state (∼2.05 eV)^[^ ^103^ ^]^ and good to excellent ^1^O_2_ generation quantum yields.^[^ ^104^ ^]^ In the ground state, **36** ^2+^ features high chemical stability, good solubility in aqueous solutions, straightforward synthesis, and high oxidation and low reduction potentials, making it a strong and versatile electron donor or acceptor for photocatalysis and photoredox chemistry in biological applications.^[^ ^10^, ^105^ ^]^ In 1999, Kodadek and coworkers first demonstrated the photo‐initiated protein cross‐linking reaction utilizing **36** ^2+^ as photocatalyst, and ammonium persulfate as the electron acceptor.^[^ ^106^ ^]^ The cross‐linked products could be obtained with very high yields under short irradiation times (<1 s). The authors proposed the following mechanism for this process: upon photoexcitation, **36** ^2+^ donated an electron to the persulfate acceptor, breaking its central O–O bond, producing Ru(III) and a sulfate radical (Figure 6a).^[^ ^107^ ^]^ Ru(III) is a strong one‐electron oxidant and oxidises Tyr residues. The resulting tyrosyl radical could form cross‐linked products through two main pathways: 1) reacting with a nearby Tyr to form arene–arene bonds, or 2) coupling with nearby nucleophilic side chains to create heteroatom–arene linkages (Figure 6a), thereby affording the modified protein.

In the above example, **36** ^2+^ was not attached to the target protein and the selectivity of the protein modification was low. Inspired by this work, Nakamura and colleagues^[^ ^108^ ^]^ introduced a novel ligand‐directed strategy for more selective protein modification utilizing local single‐electron transfer (SET) photocatalysis (**37** ^2+^).^[^ ^109^, ^110^ ^]^ This method leveraged the Ru photocatalyst to generate tyrosyl radicals on target‐selective proteins, which could then selectively be trapped by tyrosyl radical trapping agents (TRTs) via oxidative radical addition. As is shown in Figure 6b, TRT can be designed that carries a fluorescent moiety, allowing targeted protein labelling upon light irradiation.

**Figure 7:**
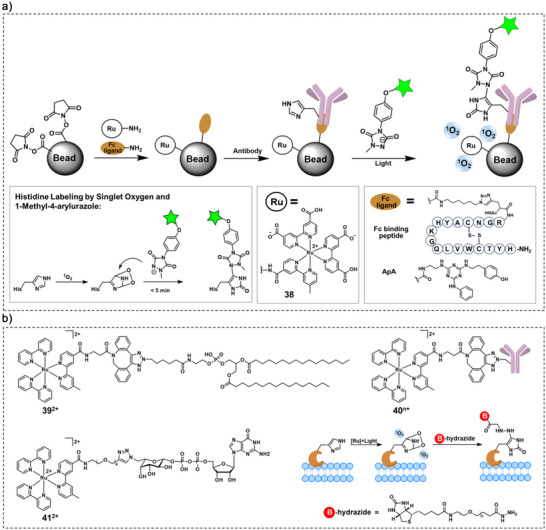
a) Scheme illustrating proximity His labelling triggered by **38**. Adapted with permission.^[^ ^111^ ^]^ Copyright 2021, American Chemical Society. b) Chemical structure of **39** ^2+^, **40** ^n+^ (the charge of protein is unknown), **41** ^2+^, and schematic illustration of the biotin‐hydrazide labelled protein on the cell surface. Adapted with permission.^[^ ^112^ ^]^ Copyright 2023, Wiley‐VCH.

Beyond Tyr, His residues have also emerged as viable targets for selective protein catalytic labelling due to their susceptibility to ^1^O_2_‐induced oxidation. The imidazole ring can undergo Diels–Alder addition with Ru‐generated ^1^O_2_ to form a reactive endoperoxide intermediate, making it a potential electrophilic site for labelling. For example, Sato et al developed a nucleophilic probe, 1‐methyl‐4‐arylurazole (MAUra), which selectively reacted with oxidised His under ^1^O_2_ generating conditions.^[^ ^111^ ^]^ By combining MAUra with a localised photocatalyst (**38**), the group achieved nanometer‐scale proximity‐driven, site‐selective His labelling, exemplified by a selective modification of Fragment crystallizable (Fc) domains on antibodies immobilised on magnetic beads (Figure 7a). Similarly, Li and coworkers have recently developed three [Ru(bpy)_3_]^2+^ derived photocatalysts carrying different cell‐anchoring moieties (**39** ^2+^, **40** *^n^* ^+^, and **41** ^2+^), which were applied to detect cell–cell interactions (CCI) with spatiotemporal resolution via a Ru‐^1^O_2_‐hydrazide system (Figure 7b).^[^ ^112^ ^]^

**Figure 8:**
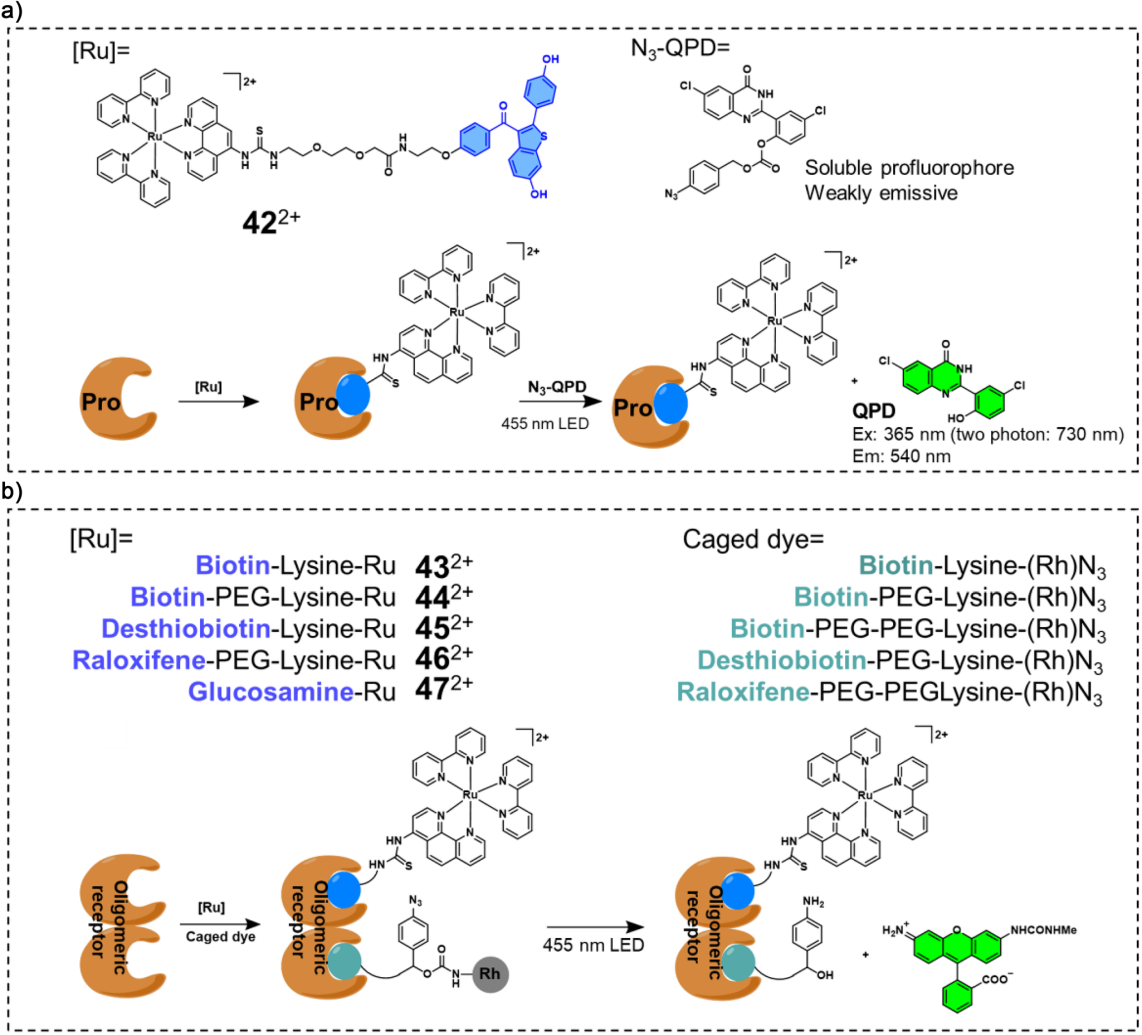
a) Chemical structure of **42** ^2+^, the pro‐fluorophore (N_3_‐QPD) and its activated product (QPD).^[^ ^113^ ^]^ b)Structure of the **43** ^2+^ **‐47** ^2+^ and caged rhodamine ligands and the schematic illustration of the photoreduction of azide‐containing immolative linkers and release of the uncaged dyes. Adapted with permission.^[^ ^115^ ^]^ Copyright 2021, American Chemical Society.

In addition to protein labelling via amino acid radical formation, an alternative strategy (type II) involves the use of Ru catalysts to unmask caged dyes (Scheme 1d). For example, Winssinger and coworkers developed a series of protein‐targeted Ru trisbipyridine complexes and combined them with the profluorophore N_3_‐QPD (Figure 8a), to achieve targeted protein labelling upon photoactivation.^[^ ^113^ ^]^ The targeting ligands could be readily designed to target the Ru complex to various endogenous proteins. As an example, the conjugate **42** ^2+^ between Ru‐trisbipyridine and raloxifene, an estrogen receptor agonist, is shown in Figure 8a. Beyond such strategies, Ru complexes have demonstrated catalytic effectiveness in a range of reactions under biologically relevant conditions, including the deprotection of a range of pro‐dyes; this field has been recently reviewed by López and Mascareñas.^[^ ^114^ ^]^

In the third strategy type (type III in Scheme 1d), both a Ru complex and a latent probe are targeted to the same protein that forms dimers or oligomers. Protein–protein interaction then allows spatial proximity between the two reactive fragments. Wissinger et al. developed a series of paired azide–rhodamine ligands and Ru‐based conjugates **43** ^2+^ **‐47** ^2+^ (Figure 8b) that achieved photoreduction of azide‐containing immolative linkers.^[^ ^115^ ^]^ This strategy enabled for example the uncaging of rhodamine fluorophores using Ru(II) conjugates in the presence of various oligomeric protein templates, inspired by nucleic acid‐templated reactions (Figure 8b).^[^ ^116^ ^]^ The generality of the approach was validated with three sets of ligands (biotin, desthiobiotin, and raloxifene) targeted to their oligomeric protein targets (streptavidin, and estrogen receptor).

The above strategy can also be used for the construction of artificial metalloenzymes (ArMs). For example, Barker and coworkers designed new ArMs by incorporating a series of [Ru(*η^6^*‐arene)(bipyridine)]^2+^ derivatives with a four‐helix bundle protein cytochrome *b*_562_, capable of catalysing the reduction of a quinolone substrate and releasing the fluorescent umbelliferone.^[^ ^117^ ^]^ As this topic is primarily applied in the construction of metalloenzymes for chemical catalysis at present, it will not be discussed in detail in this review.

Last but not least, catalytic protein labelling not involving light should also be noted. For ruthenium, the most applied catalysts in biological or biomimetic environments are Hoveyda–Grubbs Ru catalysts for olefin metathesis. These catalysts are selective toward olefins, tolerate diverse functional groups, and operate under mild aqueous conditions, which makes them ideal for introducing chemical modifications in proteins,^[^ ^118^ ^]^ for turning prodrugs into cytotoxic drugs in cells,^[^ ^119^ ^]^ or for probing morphological changes in protocells^[^ ^120^ ^]^ or biochemicals such as ethylene.^[^ ^121^ ^]^ This field has recently been reviewed by Matsuo.^[^ ^122^ ^]^

## 6 Responsive Chemosensors

Based on the unique photophysical and photochemical properties of Ru complexes, a wide range of Ru(II) complex‐based chemosensors have been designed and synthesised to selectively detect specific targets such as pH, anions, metal ions, and reactive nitrogen, oxygen, sulfur, and carbonyl species, as well as amino acids.^[^ ^123^ ^]^ Amino acids are vital to biological function, and abnormal levels can indicate diseases like cancer, neurological disorders, or metabolic conditions such as phenylketonuria.^[^ ^124^, ^125^ ^]^ In this section, we exemplified several Ru‐based chemosensors for the detection of amino acids, mainly including sulfur‐containing and His residues (Scheme 1e).

**Figure 9:**
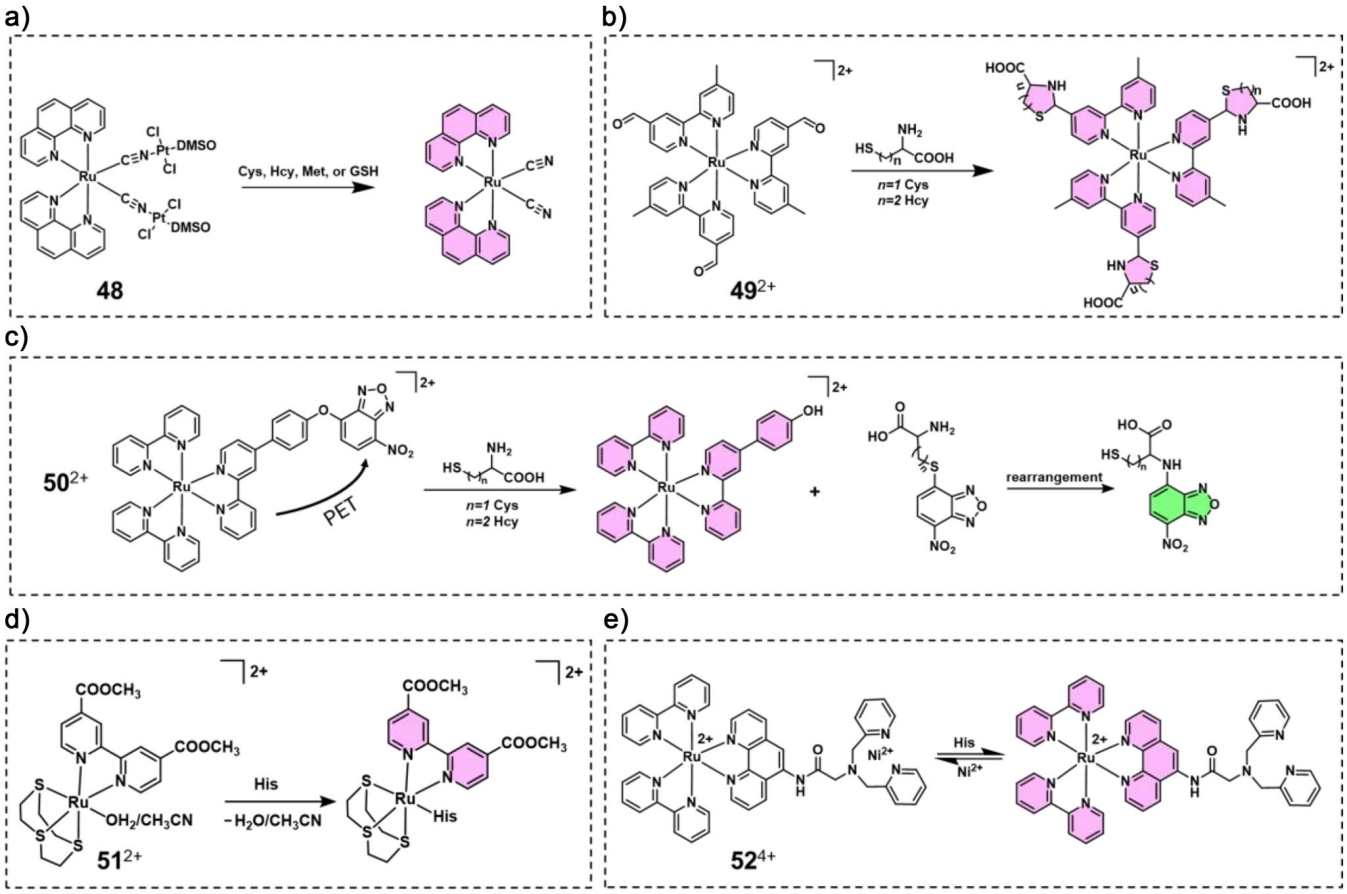
Structure of **48** a), **49** ^2+^ b), and **50** ^2+^ c) and their response to sulfur‐containing amino acids. Structure of **51** ^2+^ d) and **52** ^4+^ e) and their response to His residues.

In 2003, Lam and coworkers designed a novel luminescent chemo‐dosimeter for the selective detection of sulfur‐containing amino acids and peptides, based on a neutral trinuclear Ru(II)/Pt(II) cyano‐bridged complex cis‐Ru(phen)_2_[CN–Pt(DMSO)Cl_2_]_2_ (**48**).^[^ ^126^ ^]^ This complex exhibited quenched metal‐to‐ligand charge transfer (MLCT) emission due to electron‐withdrawing coordination of Pt(II) centre. Upon interaction with sulfur‐containing species such as Cys, homocysteine (Hcy), Met, and glutathione (GSH), the Pt(II) centres preferentially bind to the thiol/thioether groups, leading to cleavage of the Ru–CN–Pt bridges and restoration of the Ru(II) chromophore's orange‐red MLCT luminescence (Figure 9a). On the other hand, aldehyde groups can act as quenching motifs in biological sensing systems that can react with thiols to form thiazinanes and thiazolidines. For example, Yuan and coworkers,^[^ ^127^ ^]^ Chen and coworkers^[^ ^128^ ^]^ synthesised a series of Ru(II) complexes with ligands carrying aldehyde groups (**49** ^2+^ for example). The reaction of Hcy and Cys with the complexes induced significant enhancements in luminescence intensity (Figure 9b).^[^ ^128^ ^]^

In another work, Zhang and coworkers designed a quenched Ru‐based luminescent probe (Ru‐NBD) by linking a Ru(II) complex with 7‐nitro‐2,1,3‐benzoxadiazole (NBD) fluorophore through a responsive ether bond (**50** ^2+^).^[^ ^129^ ^]^ This conjugate was designed to be poorly fluorescent due to intramolecular quenching. Upon interaction with biological thiols, **50^2+^** underwent nucleophilic substitution, resulting in the release of a long‐lifetime red‐emitting Ru–phenol complex (*λ*_em _= 628 nm) and either a short‐lifetime green‐emitting NBD‐NR (in the case of Cys or Hcy, *λ*_em _= 540 nm) or a weakly emissive NBD‐SR (in the case of GSH) (Figure 9c).

His is also a potent ligand in physiological conditions, and it can be effectively detected by the coordination‐based chemosensors.^[^ ^130^ ^]^ When a Ru complex carries a weaker ligand such as H_2_O or MeCN, ligand replacement by His can happen, which will influence the coordination sphere of the metal centre, leading to a change of its physicochemical properties. For example, a Ru(II) complex bearing 1,4,7‐trithiacyclononane ([9]aneS3) and 4,49‐dimethoxycarbonyl‐2,29‐bipyridine (dcmb) ([Ru(dcmb)([9]aneS3)(CH_3_CN)]^2+^, **51** ^2+^) could be used as a luminescent switch‐on probe for His.^[^ ^131^ ^]^ It exhibited marked luminescence enhancement upon increasing the concentration of His or His‐tagged proteins and did not respond to other amino acids (Figure 9d). Alternatively, a heterobimetallic Ru(II)‐nickel(II) complex, [Ru(bpy)_2_(phen‐DPA)(Ni^2+^)]^4+^ (DPA = di(2‐picolyl)amine, **52** ^4+^) reported by Yuan and coworkers, had been a highly selective phosphorescent probe for His detection.^[^ ^132^ ^]^ The phosphorescence of the Ru(II) centre was quenched by Ni^2^⁺, while upon binding His, Ni^2^⁺ would be released, leading to a significant enhancement of ruthenium‐based emission at 603 nm (Figure 9e). More recently, Bonnet and coworkers showed that the phosphorescence of a PACT prodrug could be turned on upon light irradiation in vivo. The red emission was attributed to proximity labelling of histidine‐containing proteins by the uncaged Ru centre.^[^ ^133^ ^]^ Overall, though ruthenium compounds are versatile tools for the sensing of amino acids, their use for sensing proteins is still in its infancy.^[^ ^134^, ^135^ ^]^

## 7 Summary and Outlook

Significant progress has been made in leveraging the interaction between Ru‐based complexes and proteins for targeted cancer therapy, diagnostics, proteomic sequencing, and other biomedical applications. Recent advances include a deeper understanding of their coordination behavior with amino acids, the development of multifunctional therapeutic agents and luminophores for selective protein recognition, and their expanding utility as catalytic tools for site‐specific protein modification and labelling. At the same time, the integration of Ru complexes into emerging technologies is driving the realization of their full chemical, biological, and medicinal potential. New computational platforms such as MetalDock have been for example constructed to better predict the binding of metal complexes to proteins.^[^ ^136^ ^]^ For the local activation of anticancer Ru compounds, a recent report on sono‐induced activation opens fascinating perspectives as ultrasounds penetrate even deeper into biological tissues than NIR light.^[^ ^11^ ^]^ Ru polypyridyl complexes have also been developed in advanced super‐resolution microscopy and live‐cell confocal imaging platforms.^[^ ^137^ ^]^ In some cases, these platforms have been adapted for real‐time monitoring of protein dynamics.^[^ ^138^ ^]^ The application of Ru complexes in bioorthogonal chemistry has opened new avenues for the development of next‐generation prodrugs^[^ ^139^, ^140^ ^]^ and site‐selective protein modification.^[^ ^141^ ^]^ Altogether, these developments point to the remarkable versatility of Ru complexes and underscore their growing impact in cutting‐edge biomedical research.

Despite these advances, many applications of Ru‐based systems remain confined in vitro or to the laboratory scale. While the suggestions below only reflect the views of the authors of this minireview, we believe that further efforts are needed to translate Ru‐based molecular platforms into clinically more relevant contexts. First, the selectivity of Ru complexes may benefit from state‐of‐the‐art chemical and biological approaches used for organic compounds, such as ligands engineered with enzyme‐cleavable motifs, redox‐active amino acids, or fragments sensitive to conformational changes associated with protein aggregation. Second, directly coupling Ru complexes with serum protein scaffolds (e.g., albumin), as well as proper formulation, may improve their stability in biological media and their biodistribution without jeopardizing their reactivity, thereby facilitating translation. Third, photoactivated Ru complexes and Ru‐trisbipyridine‐based photocatalysts were initially and are still too often activated by blue light, which penetrates poorly into biological tissues. Blue light sometimes works in small mouse tumour models but remains impractical for treating larger or deeply located tumours, such as those in the brain, pancreas, or head and neck. Green‐light‐induced phototherapeutic applications of Ru complexes (e.g., TLD‐1433) are currently in clinical trial for bladder cancer,^[^ ^142^ ^]^ but in general the light absorption properties of photoactivated Ru complexes should be optimised to better absorb red or near‐infrared light (600–900 nm), thus enabling more effective light tissue penetration and hence real therapeutic applications in humans. This question has been and is still scrutinised, by the phototherapy community, but it is sometimes left aside in Ru‐based photocatalytic systems, which limits their applicability in real life. Last but not least, very little is known about the general toxicity profile and potential off‐targets of Ru complexes, while these questions become central as soon as real translation to the clinics is in sight. More knowledge is needed on the biosafety of Ru‐containing compounds, and in particular on their interactions with “anti‐targets” such as hERG or Ca_v_1.2 (cardiac toxicity),^[^ ^143^ ^]^ the P‐glycoprotein (drug‐drug interactions),^[^ ^144^ ^]^ or the serotonin receptor 5‐HT2B (valvulopathy and pulmonary hypertension),^[^ ^145^ ^]^ the blockade of which may cause potentially serious side effects in humans. As seen in the recent clinical advancements of TLD‐1433 and BOLD‐100, ruthenium complexes—despite their unique chemical and photochemical properties—must still undergo the standard safety evaluations required by the pharmaceutical industry before they can be administered to humans.

## Conflict of Interests

The authors declare no conflict of interest.

## Data Availability

Not applicable. No new data were generated or analyzed.
